# Superior Visual Timing Sensitivity in Auditory But Not Visual World Class Drum Corps Experts

**DOI:** 10.1523/ENEURO.0241-18.2018

**Published:** 2018-12-14

**Authors:** Nestor Matthews, Leslie Welch, Elena Festa

**Affiliations:** 1 Neuroscience Program, Department of Psychology, Denison University, Granville, Ohio 43023; 2 Department of Cognitive, Linguistic & Psychological Sciences, Brown University, Providence, Rhode Island 02912

**Keywords:** musical experts, perceptual learning, psychophysics, radial and rotational motion, temporal order judgments, temporal vision

## Abstract

World class drum corps require cooperation among performance artists to render precisely synchronized and asynchronized events. For example, drum corps visual aesthetics often feature salient radial and rotational motion displays from the color guard. Accordingly, extensive color guard training might predict superior visual timing sensitivity to asynchronies in radial and rotational motion displays. Less intuitively, one might instead predict superior visual timing sensitivity among world class drum corps musicians, who regularly subdivide musical tempos into brief time units. This prediction arises from the possibility that auditory training transfers cross-modally. Here, we investigated whether precise visual temporal order judgments (TOJs) more strongly align with color guard’s visual training or musicians’ auditory training. To mimic color guard visual displays, stimuli comprised bilateral plaid patterns that radiated or rotated before changing direction asynchronously. Human participants indicated whether the direction changed first on the left or right, called a TOJ. Twenty-five percussionists, 67 brass players, and 29 color guard members from a world class drum corps collectively completed 67,760 visual TOJ trials. Percussionists exhibited significantly lower TOJ thresholds than did brass players, who exhibited significantly lower TOJ thresholds than did the color guard. Group median thresholds spanned an order of magnitude, ranging between 29 ms (percussionists judging rotational asynchronies) and 290 ms (color guard judging radial asynchronies). The results suggest that visual timing can improve more by training cross-modally than intramodally, even when intramodal training and testing stimuli closely match. More broadly, pre-existing training histories can provide a unique window into the timing sensitivity of the nervous system.

## Visual Abstract

**Figure:**
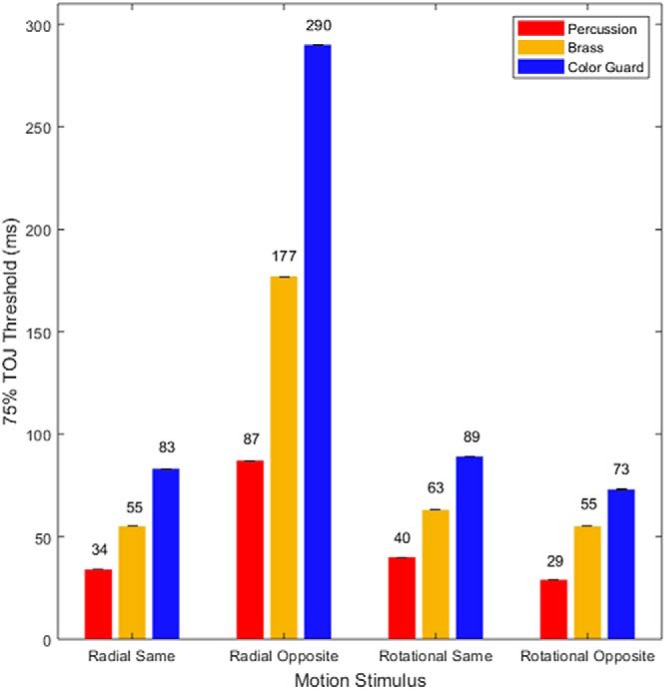

## Significance Statement

Optimal cognitive and behavioral performance typically occurs when training conditions closely match testing conditions. This fundamental principle from learning and memory predicts greater visual test performance after visual training than after auditory training. Contrastingly, a fundamental sensory principle—superior temporal precision for auditory versus visual stimuli—predicts better visual test performance after auditory training than after visual training. We observed more precise visual timing among musical experts than among visual experts who had years of visual experience that closely matched the testing stimuli. The musicians’ superior performance suggests that shared neural events mediate the precision of auditory and visual timing. Moreover, these shared neural events provide greater temporal precision than does matching sensory modalities across training and testing contexts.

## Introduction

Most vertebrate species lack the ability to synchronize their motor behavior to musical rhythms (for exceptions, see Patel et al., 2009; Hasegawa et al., 2011; Cook et al., 2013). By contrast, the human species excels at musical sensorimotor synchronization (SMS; for review, see Repp, 2005; Repp and Su, 2013). Indeed, humans almost universally exhibit rudimentary musical SMS by age 4 years (Provasi and Bobin-Bègue, 2003; Kirschner and Tomasello, 2009), and an elite few young adults achieve exquisite musical SMS.

An elite few young adults capture international attention each year with highly refined musical SMS performances during the Drum Corps International (DCI) world championship competition. Each competing corps comprises color guard, brass players and percussionists. These three groups earn points from DCI judges based on performance criteria that include the precision of musical SMS. The musical SMS features intricate melodic, harmonic, and rhythmic auditory events that arise from motor behaviors entrained to the drum major’s visually defined tempo.

**Figure 1.**
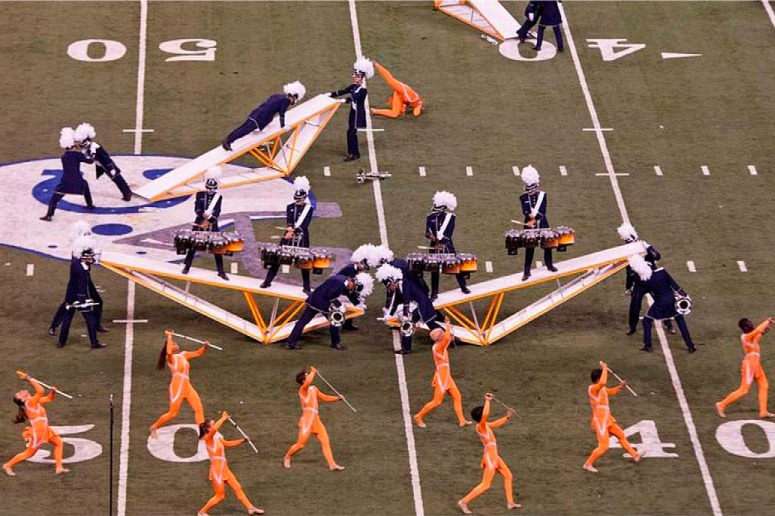
Drum corps performance. The DCI Bluecoats color guard demonstrate musical SMS via radial and rotational motion (Creative Commons Image).

DCI championship performances also feature visual displays rich in synchronized radial and rotational motion. The radial and rotational motion stem from drum corps color guard rifles, sabers, and flags twirling through the air synchronously, or at precisely timed asynchronies (Fig. 1). These radial and rotational synchronies and asynchronies become increasingly precise as color guard refine their skills for drum corps competitions. Consequently, one might expect *world class* color guard to possess exquisite sensitivity to subtle visual asynchronies in radial and rotational motion. Such visual skills could provide a unique window into the visual system’s temporal properties. This possibility inspired the present investigation of world class color guard’s visual sensitivity to asynchronies defined by radial and rotational motion.

**Figure 2.**
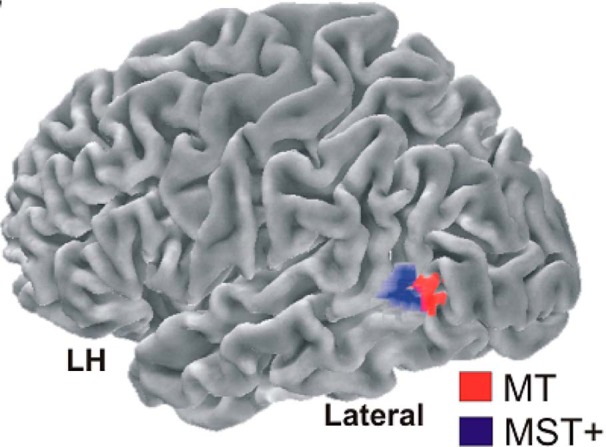
Motion physiology. Area MT (red) registers linear motion and innervates the MST^+^ complex (blue), which registers linear, radial, and rotational motion. From Pitzalis et al. (2013).

Radial and rotational motion register in the medial superior temporal (MST) brain region. Early evidence for the role of MST in radial and rotational motion came from single-cell recordings in macaque monkeys (Tanaka and Saito, 1989; Duffy and Wurtz, 1991a,b). More recent human fMRI (Smith et al., 2006; Wall et al., 2008; Strong et al., 2017) and steady-state-visual-evoked-potential (Gilmore et al., 2007) experiments similarly indicate that radial and rotational motion register in the MST+ complex (Fig. 2, blue region).

Combining MST+ plasticity and extensive color guard training predicts color guard superiority on temporal order judgments (TOJs) of asynchronies defined by radial or rotational motion. A rigorous test of this prediction entails comparing color guard to musicians within the same world class drum corps.

One might instead predict superior TOJ performance from world class drum corps musicians. Though less intuitive, this diverging prediction follows from studies demonstrating that *auditory* training enhances *visual* skills (Seitz et al., 2006; Bratzke et al., 2012; Barakat et al., 2015), and that audition dominates vision in duration perception (Ortega et al., 2014). Moreover, recent studies indicate musical training generalizes across sensory modalities (Cicchini et al., 2012; Aagten-Murphy et al., 2014). Intriguingly, among musical experts, percussionists exhibit significantly briefer visual temporal-bisection thresholds than string musicians (Cicchini et al., 2012). This difference may arise because percussionists, more frequently than other musicians, must divide musical tempos into very brief time units, such as 32nd notes. To the extent that this musical precision transfers across modalities, one might predict superior visual TOJ thresholds from world class drum corps percussionists. Recent behavioral and electrophysiological evidence suggesting a single shared clock for perception and action (e.g., producing 32nd notes) also predicts superior *visual* TOJs among percussionists (Su, 2016; Benedetto et al., 2018).

These diverging predictions framed our research question. Do precise visual TOJs more strongly align with color guard’s *visual* training or musicians’ *auditory* training? Our stimuli contained temporal asynchronies defined by radial and rotational motion, mimicking the visual displays of color guard experts. We evaluated visual TOJs in color guard, brass players, and percussionists—all from the same world class drum corps.

## Materials and Methods

The Denison University Human Participant Committee approved this study, which we conducted with the informed consent of each participant. The research adheres to the October 2008 Declaration of Helsinki. To promote reproducibility, the Open Science Framework [https://osf.io/n7gtj/] contains the complete data set and all software necessary for replicating the experiment and the statistical analyses.

### Participants

One hundred thirty-two college-aged (18–22 years old) people of either sex participated. Participants included 25 percussionists, 71 brass players, and 36 color guard from the DCI Bluecoats, a highly competitive music and dance ensemble.

All participants shared several important experiences. After passing a rigorous audition to earn membership into the Bluecoats, all members participated together in 5 weeks of spring training, 7 d/week. A typical spring training rehearsal day lasted 13 h. Most rehearsal days comprised shared physical training, morning and afternoon sectional rehearsals, and concluded with the entire group practicing as one ensemble. The entire ensemble typically ate four meals together (breakfast, lunch, and dinners before and after evening ensemble rehearsals) daily and observed the same sleep curfew. All participants entered the present experiment after completing their shared evening ensemble rehearsal, but before their shared sleep curfew.

The three groups (percussion, brass, and color guard) also earned similar ranks in the DCI 2017 world championship competition, which occurred 2 months after our study. The rankings came from DCI championship judges who had no affiliation with or knowledge of our study. Relative to other world class DCI percussionists, our percussionists earned a fifth place ranking from one DCI judge, and a sixth place ranking from another. Relative to other world class DCI brass players, our brass players ranked in fourth place. And, relative to other world class DCI color guards, our color guard ranked in fifth place. For context, those rankings occurred among 24 world class (and an additional 22 lower-ranked “open class”) DCI drum corps participating in the 2017 world championship. In short, our three groups (percussion, brass, color guard) shared similar spring training experiences, and earned on their respective tasks comparable ranks from independent judges. The shared experiences and similar ranks rendered the groups effective controls for each other.

In addition to their shared experiences and similar DCI rankings, the groups also exhibited relevant similarities, and relevant differences, in their training histories. The training histories appear in cells in Table 1, which each contain two numbers: training prevalence, followed by training duration. Training prevalence refers to the number of group members that reported having any training experience in a given category: percussion; brass; spinning; or other instrument. Training duration refers to the median number of training years among those who reported any training experience within a category. Visually inspecting Table 1 reveals the task-specific training history of each group, both in prevalence and duration. For example, the prevalence and duration of the percussionist’s percussion training exceeded that of the other groups and the other training categories. Correspondingly, the prevalence and duration of the brass players’ brass training exceeded that of the other groups and training categories. Likewise, the prevalence and duration of the color guard’s spinning training exceeded that of the other groups and training categories. Despite this group specificity, the groups exhibited comparable prevalence and durations within their respective specialties. Regarding prevalence, all percussionists (25 of 25), all brass players (67 of 67), and all color guard members (29 of 29) reported training on their respective tasks. Regarding the median training durations on their respective tasks, percussionists reported 8 years, brass players reported 8 years, and color guard members reported 7 years. Thus, the training histories indicate quantitative group similarities (prevalence and duration), but qualitative group differences (percussion vs brass vs color guard). This combination afforded an opportunity to examine how comparable numbers of different training types might affect visual timing sensitivity.

**Table 1:** Training history

Training history	Percussionists (*n* = 25)	Brass players (*n* = 67)	Color guard members (*n* = 29)
Percussion	25, 8	10, 2	2, 4
Brass	3, 2	67, 8	0, n/a
Spinning	0, n/a	6, 1	29, 7
Other Instrument	8, 9	34, 7.5	7, 4

The first number in each cell indicates the training prevalence (i.e., the number of group members who reported any training experience on a given activity). The second number in each cell reflects the median years of training among the group members who reported any training experience on a given activity. The three groups reported comparable training prevalence (100%) and training durations (7–8 years) on their respective specialties. By contrast, each group reported relatively higher training prevalence and longer training durations on the activity specific to their group. n/a, Not applicable.

To summarize, the three participant groups matched each other well on several variables. These include age (18–22 years), daily spring training schedule, eating and sleeping schedules, DCI rank, training prevalence, and training duration on their respective tasks. The groups differed markedly on the type of training (percussion vs brass vs color guard).

### Materials and apparatus

The experiment ran on HP EliteOne 800 desktop computers, each with a Microsoft Windows 10 Enterprise operating system. Matlab 2017a software called functions from the Psychophysics toolbox (Brainard, 1997; Pelli, 1997). We set the spatial resolution of the 23 inch flat-screen HP LCD displays to 1920 × 1080 pixels, and set the vertical refresh rate of the display to 60 Hz. Although we did not stabilize head position, participants typically viewed the monitor from ∼57 cm.

### Plaid stimuli

**Figure 3.**
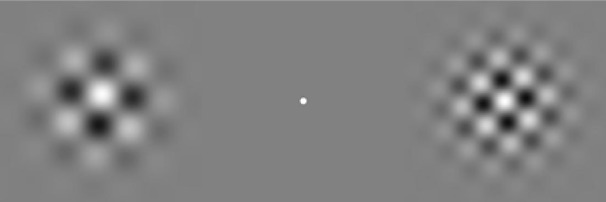
Sample movie frame. On each trial, a pair of bilaterally presented plaids either radiated or rotated. The plaids within each pair moved initially in either the same direction or opposite directions, randomly across trials. The two plaids subsequently changed direction, either at the same time or at various asynchronies. Participants indicated whether the left plaid or right plaid changed direction first—a TOJ.

On each trial, participants viewed bilaterally presented dynamic plaid stimuli, shown in a 1 s (60 frame) movie. A sample frame from one movie appears in Figure 3. We centered each plaid 9.7° left or right of a white fixation point (152 cd/m^2^) in a gray surround (32.5 cd/m^2^). Each plaid had 95.47% Michelson contrast, a two-dimensional Gaussian window, and a 9.7° diameter. Within each plaid, the two component gratings had identical spatial frequencies and identical spatial phases. The component spatial frequencies ranged randomly across ∼3 octaves (0.25–1.9 cycles/°), and the spatial phases ranged randomly across 360°. One of the two component orientations on each trial ranged randomly across 180°, and the other differed by 90° from that randomly selected orientation.

### Radial and rotational speeds and direction changes

On each trial, the left and right plaid-component gratings either shared a radial speed of 2 octaves/s or shared a rotational speed of 0.102 rotations/s. Those radial and rotational speeds generated comparable local linear speed gradients, which ranged between 0 and 3.114°/s. Each plaid initially moved in one radial or rotational direction for 333–666 ms, then reversed to the opposite direction for the remaining 333–666 ms. The direction reversals in the left and right plaids occurred either synchronously, or at ±67, ±133, or ±200 ms asynchronies. For illustration and ease of viewing, Movies 1–8 display 300 ms asynchronies for each of the stimulus conditions described in Table 2.

**Table 2:** Sample dynamic plaid stimuli

Movie	Motion type	Initial directions	First direction reversal
1	Radial	Same	Left
2	Radial	Same	Right
3	Radial	Opposite	Left
4	Radial	Opposite	Right
5	Rotational	Same	Left
6	Rotational	Same	Right
7	Rotational	Opposite	Left
8	Rotational	Opposite	Right

Movies 1–8 display the various stimulus conditions, with 300 ms temporal asynchronies for illustration.

Movie 1. Sample radial stimuli moving in the same initial direction, with the left side reversing direction first. 10.1523/ENEURO.0241-18.2018.video.1

Movie 2. Sample radial stimuli moving in the same initial direction, with the right side reversing direction first. 10.1523/ENEURO.0241-18.2018.video.2

Movie 3. Sample radial stimuli moving in opposite initial directions, with the left side reversing direction first. 10.1523/ENEURO.0241-18.2018.video.3

Movie 4. Sample radial stimuli moving in opposite initial directions, with the right side reversing direction first. 10.1523/ENEURO.0241-18.2018.video.4

Movie 5. Sample rotational stimuli moving in the same initial direction, with the left side reversing direction first. 10.1523/ENEURO.0241-18.2018.video.5

Movie 6. Sample rotational stimuli moving in the same initial direction, with the right side reversing direction first. 10.1523/ENEURO.0241-18.2018.video.6

Movie 7. Sample rotational stimuli moving in opposite initial directions, with the left side reversing direction first. 10.1523/ENEURO.0241-18.2018.video.7

Movie 8. Sample rotational stimuli moving in opposite initial directions, with the right side reversing direction first. 10.1523/ENEURO.0241-18.2018.video.8

### Task

On each trial, participants pressed either the left or right arrow key on a standard keyboard to signal whether the left or right plaid changed direction first, called a TOJ. Immediate accuracy feedback followed each response.

### Procedure

To develop familiarity with the dynamic plaid stimuli, participants began the experiment passively viewing Movies 1–8, which contain relatively exaggerated (300 ms) asynchronies. Participants then completed a practice block of 112 TOJ trials, separated into four block-randomized 28 trial sets. Each 28 trial set comprised two motion types (radial and rotational) crossed with two initial directions (same and opposite) and seven temporal asynchronies (−200, −133, −67, 0, 67, 133, and 200 ms). Negative (“left-lagging”) and positive (“left-leading”) asynchronies corresponded respectively to trials on which the right plaid and left plaid changed direction first. When the left and right plaids changed direction simultaneously (0 asynchrony trials) the computer predesignated the correct response as “left first” or “right first” with equal probability. This neutral feedback on 0 asynchrony trials allowed us to assess each participant’s left-first versus right-first TOJ response bias (Matthews et al., 2013).

After the 112 trial practice block, participants completed five additional 112 trial blocks (560 total trials) for analysis. These 112 trial blocks matched the practice block in all ways, including the 4 block-randomized 28 trial sets. Between the 112 trial blocks, each participant rested for 30 s while the computer displayed the participant’s cumulative percentage of correct responses. The experiment typically required ∼ 25 min and began at approximately 10:15 P.M., after all participants had completed a 13 h drum corps rehearsal. Last, on finishing the psychophysical experiment participants completed a training history questionnaire [available on the Open Science Framework (https://osf.io/n7gtj/)], which generated the summary in Table 1.

### Research design

We administered the independent variables via a 3 × 2 × 2 (group × motion type × initial directions) mixed quasi-experimental research design. The quasi-experimental designation reflects nonrandom assignment to levels of the between-participant group variable, based on each participant’s pre-existing expertise in percussion, brass, or color guard. By contrast, and as noted above, we used block randomization to directly manipulate the following within-participant independent variables: motion type (radial, rotational) and initial directions (same, opposite). Control variables included counterbalancing which plaid (left or right) changed direction first, and the temporal asynchrony of the direction changes (−200, −133, −67, 0, 67, 133, and 200 ms). The dependent variables included the precision and speed of each participant’s TOJs.

### Temporal asynchronies and typical color guard timing demands

**Figure 4.**
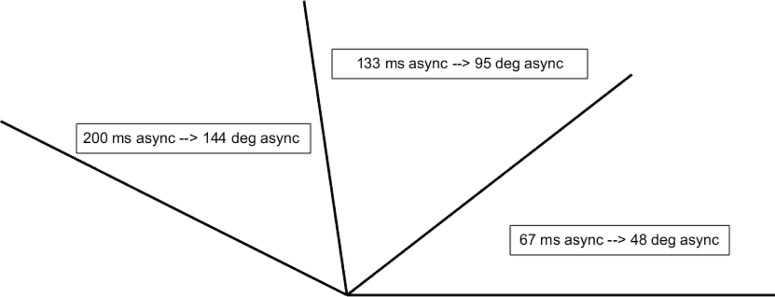
Temporal asynchronies and color guard timing demands. The 0, 67, 133, and 200 ms asynchronies tested here correspond to 0°, 48°, 95°, and 144° rotational asynchronies in typical color guard performance contexts. In this example, at the 0 ms asynchrony all rifles would align horizontally with “tips to the right.” One would expect a world class color guard to show superior sensitivity across a wide range of spatiotemporal asynchronies.

During color guard training, adjacent rifles must precisely synchronize. Accordingly, one would expect color guard experts to demonstrate superior sensitivity even to very small asynchronies. The temporal asynchronies between our left and right stimuli pertain to typical color guard timing demands in the following way. During performances, color guard rifles often rotate synchronously with a marching tempo of 120 beats/min. This corresponds to a full 360° rotation every 500 ms, or 0.72°/ms. At that rotational speed, our 0, 67, 133, and 200 ms temporal asynchronies would generate 0°, 48°, 95°, and 144° angular mismatches between adjacent rifles. Figure 4 schematizes the spatial asynchronies (rifle-tip-to-rifle-tip angular discrepancies) that our temporal asynchronies would create in typically occurring color guard contexts. Stated differently, the angles in Figure 4 intuitively represent a space–time relationship; longer asynchronies equate to greater mismatches between rotating color guard rifles. Notably, the 144º spatial (angular) mismatch arising from a 200 ms temporal asynchrony approaches the largest possible angular mismatch: 180º (i.e., antiphase). Antiphase (i.e., one rifle pointing rightward while another points leftward) would occur at a 250 ms temporal asynchrony, under typical color guard timing demands. Consequently, one would expect world class color guard to exhibit superior sensitivity to asynchronies sampled within the 0–250 ms (0–180°) range. The present asynchronies represent samples from that range.

### Motion stimuli and typical color guard retinal motion

The present motion stimuli matched the color guard’s visual experiences in important ways. First, radial and rotational motion typify the rising (radially contracting), falling (radially expanding), and spinning (rotating) rifles, sabers, and flags in color guard displays. Second, our “same” and “opposite” direction conditions correspond to the color guard’s synchronized and precisely asynchronized motion displays. Specifically, their synchronized displays parallel our same direction condition. For example, rifles that rise together generate synchronized radial contraction on the retina. Rifles that fall together generate synchronized radial expansion on the retina. By contrast, opposite directions of radial motion (retinal expansion vs retinal contraction) occur when neighboring color guard members toss their rifles skyward in antiphase. As one rifle rises (peripherally contracts), another rifle falls (peripherally expands), creating a visual domino effect for the audience. In this way, our same and opposite radial conditions correspond to the color guard’s synchronized and precisely asynchronized radial motion displays. A similar principle holds for rotational motion, as the color guard flags spin in either the same initial directions or opposite initial directions.

### Statistical analyses

#### Signal detection theory

For each participant, we used standard procedures from signal detection theory (Green and Swets, 1966) to evaluate time sensitivity i.e., TOJ precision indexed by *d*´. Operationally, hits and false alarms occurred, respectively, when participants made left-first responses and the left plaid changed direction first or second. Computationally, we determined each participant’s *d*´ value using the formula *d*´ = *Z*_Hits_ − *Z*_FalseAlarms_, with the *Z*-distribution SD = 0.5. Accordingly, *d*´ = 0.67 corresponded to nonbiased 75% correct performance.

For each participant, we computed 16 *d*´ values. Twelve indexed precision at each (2 × 2 × 3) combination of motion type (radial, rotational), initial-directions (same, different), and nonzero asynchrony magnitude (67, 133, and 200 ms). Four others pertained again to each motion type and initial-directions combination, but after pooling across negative asynchronies to determine *Z*_FalseAlarms_ and positive asynchronies to determine *Z*_Hits_. Because *z*-transformations require proportions greater than zero and less than one, we adopted the following procedure from Stanislaw and Todorov (1999). For participants achieving zero false alarms, we assumed 0.5 of 20 or 0.5 of 60 false alarms, respectively, when evaluating individual versus pooled temporal asynchronies. Conversely, for participants achieving 100% hits, we assumed 19.5 of 20 or 59.5 of 60 hits, respectively, when evaluating individual versus pooled temporal asynchronies. Note that pooling temporal asynchronies to estimate TOJ sensitivity (*d*´) parallels how a psychometric function pools temporal asynchronies to estimate TOJ thresholds.

#### Psychometric functions and thresholds

We further evaluated group differences in TOJ precision by constructing 12 psychometric functions, one for each (2 × 2 × 3) combination of motion type, initial directions, and group. For a given combination of motion type and initial directions, the ordinate of the psychometric function reflected the median proportion of left-first responses of a given group. The abscissa comprised the seven asynchronies, which ranged between −200 ms (“left lagging”) and +200 ms (“left leading”), in ∼67 ms steps. A least-squares procedure fit the data with the following sigmoidal function:

$$\frac{1}{1+exp(-K\left(X-Xo\right))}$$

*K* and *Xo* determine the slope and midpoint, respectively, of the sigmoid. In each case, Pearson correlations indicated that the sigmoid significantly fit (*p* < 0.00001) and explained >97% of the response variability. The significant sigmoidal fits permitted estimating the 75% just noticeable difference (i.e., TOJ thresholds). We defined each TOJ threshold as half of the stimulus change (temporal asynchrony) required to alter the left-first response rate from 0.25 to 0.75. Lower thresholds indicate better time sensitivity (i.e., finer TOJ precision).

### Nonparametric Monte Carlo simulations

We statistically evaluated *d*´ using nonparametric Monte Carlo simulations. The need for this nonparametric approach arose when Lilliefors tests demonstrated that data from several experimental conditions failed to satisfy the normalcy assumption underlying ANOVAs.

One series of nonparametric Monte Carlo simulations evaluated pairwise group differences in *d*´, indexed by TOJ precision. These pairwise group comparisons included percussion versus brass, brass versus color guard, and percussion versus color guard. We crossed each of those three pairwise group comparisons with two motion types, two initial directions, and four asynchronies (67, 133, and 200 ms, and their aggregate). For each combination of those variables, the computer generated 10,000 simulations by randomly shuffling the group designations before calculating a simulated “median group difference” in *d*´. The computer then sorted the 10,000 simulated median–group differences from low to high and evaluated where the empirically observed median–group difference ranked within this simulated distribution. Statistically significant differences occurred when the empirically observed median–group difference exceeded the 95th percentile of the simulated distribution. Larger median *d*´ differences reflect larger effect sizes.

A different series of nonparametric Monte Carlo simulations evaluated the extent to which motion type and initial directions interacted to influence time sensitivity, indexed by TOJ precision (*d*´). This series paralleled the above-described series by again generating a context of 10,000 Monte Carlo median *d*´-based simulations. Here, though, the median *d*´-based simulations arose from the following 2 × 2 interaction computation: (Radial Same *d*´ − Radial Opposite *d*´) − (Rotational Same *d*´ − Rotational Opposite *d*´).

Using that computation, the computer generated 10,000 simulated “interactions” after randomly shuffling each participant’s *d*´ values across the radial-same, radial-opposite, rotational-same, and rotational-opposite conditions. (Note that shuffling the *d*´ values in this within-participant manner exactly retained the empirically observed consistent individual differences on each of the 10,000 simulations.) The computer then sorted the 10,000 simulated interactions from low to high, and evaluated where the empirically observed interaction ranked within this simulated distribution. Statistically significant interactions occurred when the empirically observed interaction exceeded the 95th percentile of the simulated distribution. Larger median *d*´-based interactions reflect larger effect sizes. We repeated this procedure separately for each group and asynchrony combination, and after averaging across groups and asynchronies.

As noted above, the Open Science Framework (https://osf.io/n7gtj/) contains the data and software for conducting the Monte Carlo simulations.

#### Reaction time

The Matlab software that controlled the experiment also measured the reaction time (RT) associated with each response. The reaction time clock began with the offset of the dynamic-plaid stimuli and ended with the participant’s response on either the left or right arrow key. For each participant, we computed the median reaction time in 28 separate experimental conditions. These 28 (2 × 2 × 7) included two motion types (radial, rotational) crossed with two initial directions (same, opposite) and seven asynchronies (−200, −133, −67, 0, 67, 133, and 200 ms). Within each of the 28 conditions, we determined the participants’ median reaction time separately for each group (percussion, brass, color guard). The group median reaction times provided additional information about the extent to which percussion, brass, and color guard members differed on TOJs. The reaction time data also provided a measure for evaluating possible trade-offs between the speed and precision of responding.

### Inclusion/exclusion criteria

The statistical analyses included data from each participant whose TOJ performance statistically exceeded chance (binomial test, *p* < 0.001). This inclusion criterion minimally required 57.5% correct TOJ performance across the 480 nonzero asynchrony trials for analysis. (The remaining 80 zero-asynchrony trials for analysis provided no basis for objectively determining a correct TOJ.) This performance criterion resulted in excluding data from 11 participants (8.33% of 132 participants). Data from the remaining 121 participants (91.67% of 132 participants) appear in the Results. As noted above, the Open Science Framework [https://osf.io/n7gtj/] contains the raw data from all 132 participants.

## Results

### Group differences in *d*´

**Figure 5.**
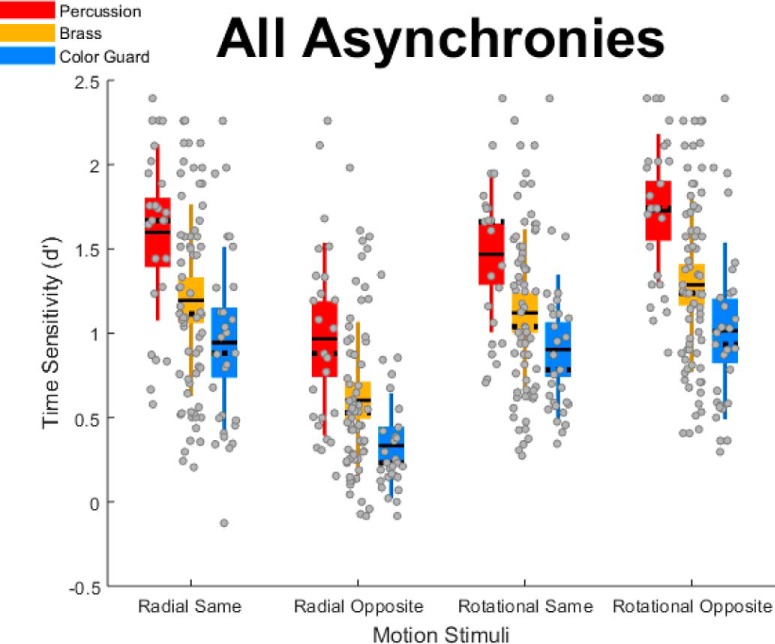
Group differences in time sensitivity aggregated across all asynchronies; descriptive statistics. The graph shows *d*´—indexed by TOJs—separately for the four motion stimulus conditions. Higher *d*´ values indicate better performance. Red, gold, and blue boxes, respectively, reflect percussion, brass, and color guard. Unlike standard box plots, each box extends upward and downward by 1 SD from the mean—the solid horizontal line centered within each box. The dotted horizontal line indicates the median, and the error bars indicate the 95% confidence interval. Gray dots correspond to individual data points.

Figure 5 illustrates our main finding, group differences in *d*´. Specifically, percussionists (Fig. 5, red boxes) exhibited the greatest time sensitivity, followed by brass (Fig. 5, gold boxes), then the color guard (Fig. 5, blue boxes). This pattern occurred across the four motion conditions (radial same direction; radial opposite direction; rotational same direction; rotational opposite direction). The pattern also occurred at each of the three temporal asynchronies (67, 133, and 200 ms), displayed in separate frames of Movie 9.

Movie 9. Group differences in time sensitivity at various asynchronies; descriptive statistics. The movie’s four frames successively depict *d*´ at the averaged, 67, 133, and 200 ms asynchronies, each across the four motion types. Higher *d*´ values indicate better performance. Red, gold, and blue boxes respectively reflect percussion, brass, and color guard. Unlike standard box plots, each box extends upward and downward by one standard deviation from the mean –the solid horizontal line centered within each box. The dotted horizontal line indicates the median, and the error bars indicate the 95% confidence interval. Gray dots correspond to individual data points that collectively satisfied the normalcy assumption (Lilliefors test). Gray crosses correspond to individual data points that collectively failed to satisfy the normalcy assumption (Lilliefors test). 10.1523/ENEURO.0241-18.2018.video.9

We used the data in Figure 5 to generate the statistical analyses summarized in Table 3. Within each motion condition, the percussionists’ median *d*´ value significantly exceeded that of brass players, whose median *d*´ value significantly exceeded that of the color guard. As described in Materials and Methods, we evaluated these differences using Monte Carlo simulations on group medians rather than on group means. This choice reflects the fact that data in some conditions failed to satisfy the normalcy assumption, per Lilliefors test. Although we computed the group differences in Table 3 after aggregating across the three asynchronies (67, 133, and 200 ms), a similar group difference pattern generally occurred within each asynchrony. The one exception to this group difference pattern occurred in the rotational opposite 200 ms asynchrony. In that condition, percussionists and brass players generated comparable median *d*´ values as performance approached the ceiling (Movie 9, final frame).

**Table 3.** Group differences in time sensitivity aggregated across all asynchronies; inferential statistics

Motion condition	Group comparison	Median difference (*d*´)	Monte Carlo *p* value
Radial same	Percussion vs brass	0.5530	0.0031
	Brass vs color guard	0.2323	0.0447
	Percussion vs color guard	0.7853	0.0006
Radial opposite	Percussion vs brass	0.3494	0.0188
	Brass vs color guard	0.2980	0.0002
	Percussion vs color guard	0.6474	<0.0001
Rotational same	Percussion vs brass	0.6208	0.0001
	Brass vs color guard	0.2556	0.0397
	Percussion vs color guard	0.8764	<0.0001
Rotational opposite	Percussion vs brass	0.5027	0.0033
	Brass vs color guard	0.3035	0.0216
	Percussion vs color guard	0.8062	<0.0001

In each motion condition, percussion significantly exceeded brass and brass significantly exceeded color guard in time sensitivity, indexed by group differences between median *d*´ values. Larger median *d*´ differences reflect larger effect sizes.

### Group differences in psychometric functions and thresholds

**Figure 6.**
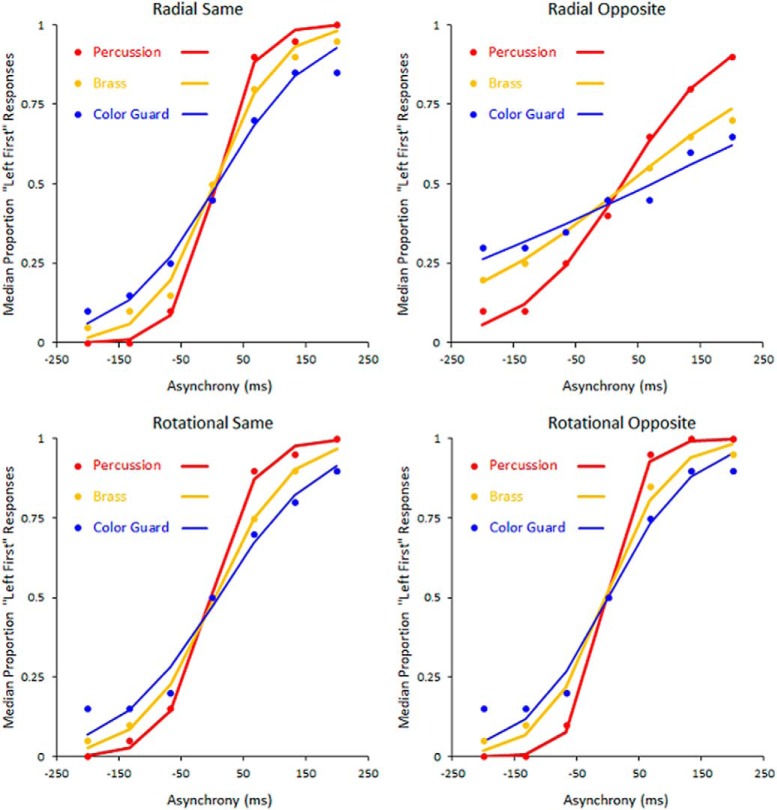
Psychometric Functions. Red, gold, and blue dots indicate the median proportions of left-first responses from percussion, brass, and color guard members, respectively. Trend lines indicate the best fitting psychometric functions. Steeper slopes reflect better temporal precision (lower thresholds). In each of the four motion stimulus conditions (shown in separate panels), percussionists exhibited the steepest slope, followed by brass players, then color guard.

Figure 6 shows psychometric functions. Each tracks left-first responses as the direction-defined change on the left followed (negative asynchronies) or preceded (positive asynchronies) that on the right. Panels correspond to the radial-same, radial-opposite, rotational-same, and rotational-opposite conditions. Within each panel, percussionists (red) generated the steepest slope, brass players (gold) generated an intermediate slope, and the color guard (blue) generated the shallowest slope. Despite these group differences in slope (precision), no group differences emerged in the midpoints (accuracy) of psychometric functions. Moreover, all midpoints tended toward the 0 ms asynchrony. This indicates that participants made left-first and right-first responses in a nonbiased manner.

**Figure 7.**
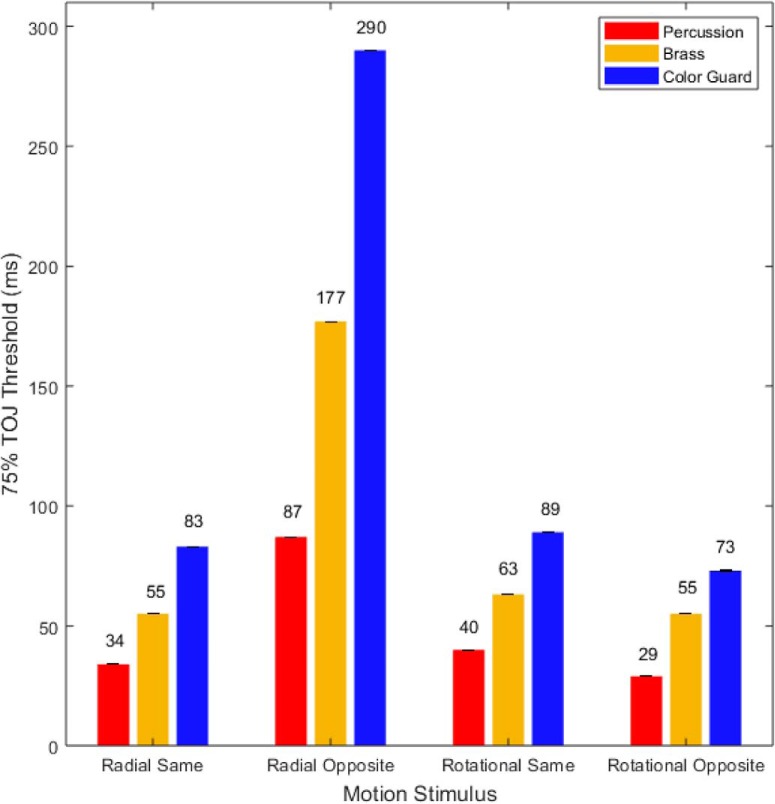
The 75% TOJ thresholds (in milliseconds). Red, gold, and blue bars indicate the 75% TOJ thresholds in milliseconds for the percussion, brass, and color guard members, respectively. Lower thresholds indicate better performance. In each of the four motion stimulus conditions, percussionists exhibited the lowest thresholds, followed by brass players, then by color guard members. The radial-opposite motion stimuli more than doubled thresholds for each group. Error bars (too small to be visible in some conditions) reflect 1 SE from the threshold estimate of the psychometric function.

The psychometric functions in Figure 6 provided the basis for the TOJ threshold estimates in Figure 7. In each of the four motion conditions, percussionists (Fig. 7, red) exhibited the lowest thresholds (finest precision). In turn, brass players (Fig. 7, gold) exhibited intermediate thresholds, and the color guard (Fig. 7, blue) exhibited the largest thresholds (lowest precision). The thresholds spanned an order of magnitude, ranging between 29 ms (rotational opposite motion, percussion) and 290 ms (radial opposite motion, color guard). For context, one can compare each of the thresholds in Figure 7 to the durations in Table 4, which displays various fractions of a musical beat at tempos commonly used in drum corps performances. As an example, the percussionists’ finest TOJ threshold (29 ms; 34.48 Hz) reflects a temporal interval briefer than that between 32nd notes played at 180 beats/min (41.67 ms; 24 Hz). At the other extreme, the color guard’s largest TOJ threshold (290 ms; 3.44 Hz) reflects a temporal interval longer than that between 8th notes played at 120 beats/min (250 ms; 4 Hz). This comparison highlights the visual time sensitivity differences between percussionists and color guard in musical contexts (tempos) that typify DCI performances.

**Table 4.** Musical note durations and temporal frequencies at common drum corps tempos

	Tempo = 180 beats/min	Tempo = 120 beats/min
Beats/s	3	2
Milliseconds per beat (quarter note)	333.33	500
Milliseconds per 8th note	166.67	250
Milliseconds per 16th note	83.33	125
Milliseconds per 32nd note	41.67	62.5
TF in Hz per beat (quarter note)	3	2
TF in Hz per 8th note	6	4
TF in Hz per 16th note	12	8
TF in Hz per 32nd note	24	16

In the rotational opposite motion condition percussionists exhibited a temporal grain finer than 32nd notes at 180 beats/min. By contrast, in the radial opposite motion condition, color guard exhibited a temporal grain slower than 8th notes at 120 beats/min.

### Group differences in meeting the inclusion criterion

**Figure 8.**
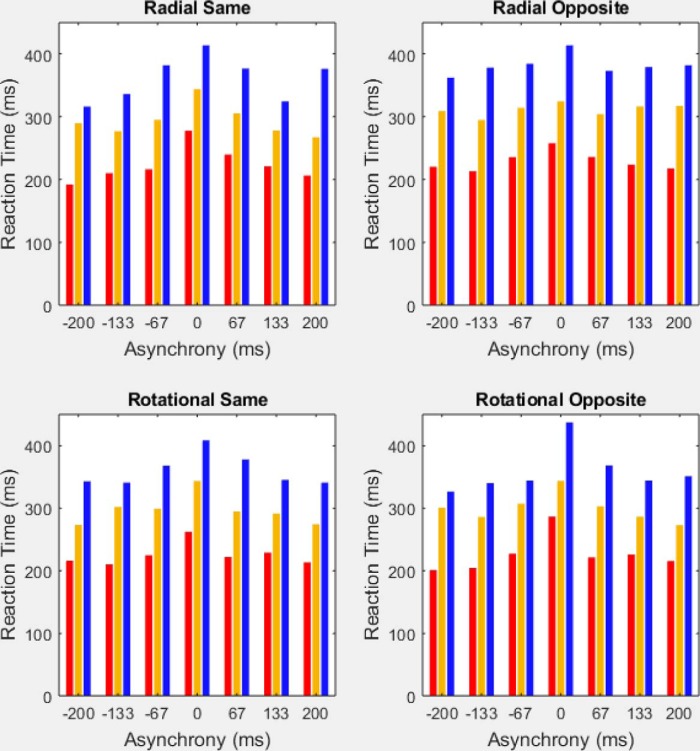
Median reaction times by group across all 28 experimental conditions. The four panels depict median reaction times in the radial-same, radial-opposite, rotational-same, and rotational-opposite motion conditions, each at seven TOJ asynchronies. In all 28 experimental conditions, percussionists (red) exhibited the fastest RTs, followed by brass players (gold), then color guard members (blue). For each motion condition and group, reaction times slowed (increased) at the zero asynchrony (i.e., when the stimuli provided no information for TOJs).

Group differences in meeting the inclusion criterion also paralleled the group differences in time sensitivity. The highest percentage of included participants occurred among percussionists (100%; 25/25), followed by brass players (94.4%; 67/71) then color guard (80.5%; 29/36). Additional context arises when considering the 10.4 percentage-point standard deviation associated with the percentage-correct distribution from all participants who met the 57.5% correct inclusion criterion. Notably, even the lowest performing percussionist scored 72.2% correct, i.e., *more* than an SD (SD = 10.4 percentage points) above the inclusion criterion. Dissimilarly, 8 of 67 brass players and eight of 29 color guard met the inclusion criterion by *less* than one standard deviation (SD = 10.4 percentage points). In short, the exclusion criteria differentially affected the groups, removing more low-performing color guard than brass players, and removing more low-performing brass players than percussionists. Consequently, including data from all participants would have generated larger group differences in time sensitivity than those displayed in Figures 6, 7, and 8.

### Group differences in reaction time

The same pattern of group differences occurred also for RT, shown in Figure 8. Figure 8 comprises four panels, one for each of the motion conditions (radial same direction; radial opposite direction; rotational same direction; and rotational opposite direction). The graph for each motion condition displays the median reaction time of each group at each of our seven TOJ asynchronies. Across all (4 motion conditions × 7 asynchronies) 28 experimental conditions, percussionists (Fig. 8, red bars) exhibited the fastest (lowest) median reaction times, followed by brass (Fig. 8, gold bars), then the color guard (Fig. 8, blue bars). The chance probability of a given three-group sequence (e.g., percussion < brass < color guard) occurring in all 28 experimental conditions equals 1/6^28^ (*p* < 1.63^−22^).

Table 5 further summarizes group RT differences. The values in Table 5 reflect the following computation. For each of the three groups, we listed the median reaction time in each of the (4 motion conditions × 7 asynchronies) 28 experimental conditions. We then computed the three below-described lists of 28 pairwise difference scores—one difference score for each experimental condition.

**Table 5.** Group differences in median RTs

RT differences (ms)	Percussion–brass	Brass–color guard	Percussion–color guard
Median	−74	−66	−138
Minimum	−57	−26	−103
Maximum	−100	−109	−170

Values reflect the median, smallest, and largest group differences in median RTs (in ms) observed across 28 experimental conditions.

List 1: Percussion median RTs minus Brass median RTs.

List 2: Brass median RTs minus Color Guard median RTs.

List 3: Percussion median RTs minus Color Guard median RTs.

For each of these three difference score lists (Table 5, columns) we identified the median, maximum, and minimum RT difference scores (Table 5, rows). For context, the percussionists’ minimum reaction time advantage over brass (57 ms) corresponds to an interval longer than that between 32nd notes played at 180 beats/min (41.67 ms; 24 Hz). At the other extreme, the percussionists’ maximum reaction time advantage over color guard (170 ms) corresponds to an interval longer than that between 8th notes played at 180 beats/min (166.67 ms; 6 Hz).

The reaction time data place an important constraint on interpreting group differences in time sensitivity. Specifically, the reaction time data argue against mere trade-offs between the speed and precision of the participants’ TOJs. Moreover, the reaction time and temporal precision (*d*´) data independently demonstrate an identical TOJ performance pattern among ensembles within the same drum corps: percussion then brass then color guard.

Despite the group differences in median reaction time, all groups exhibited greater median reaction times at the zero asynchrony than at nonzero asynchronies. Indeed, visually inspecting Figure 8 reveals that these slower reaction times at the zero asynchrony occurred in each of the four motion conditions. This effect matches that observed in a previous TOJ study that attributed the slowed reaction times at the zero asynchrony to TOJ decision space uncertainty (Matthews et al., 2016).

### Correlation between reaction time and *d*´

**Figure 9.**
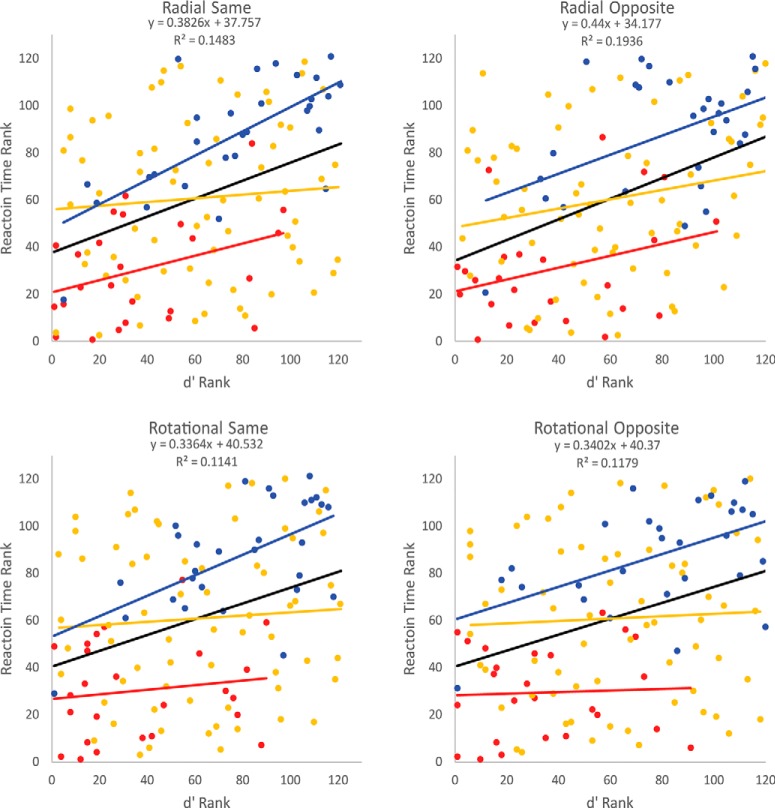
Reaction time by *d*´ scatter plots. The four scatter plots correspond to the four motion–stimulus conditions. Each plot contains rank-transformed data from all 121 participants who met the inclusion criterion. Black trendlines, equations, and *R* ^2^ values indicate significant (*p* < 0.001) positive correlations between the reaction time rank and *d*´ rank on the TOJ task. Within each plot percussionists (red dots, red long-dashed trendlines) tended toward the bottom left corner, indicating superior performance on each variable. Brass players (gold dots, medium-dashed trendlines) generally ranked intermediately, and color guard members (blue dots, blue short-dashed trendlines) tended to rank less favorably (top right corner).

A reviewer inquired about whether reaction time and *d*´ correlated on a participant-by-participant basis. The scatter plots in Figure 9 address this correlation, separately for the radial-same, radial-opposite, rotational-same, and rotational-opposite conditions. Because data in some conditions failed to satisfy the normalcy assumption, we rank transformed the reaction time and *d*´ data for each participant in each condition. Within each condition, the black trendline, equation, and *R* ^2^ value indicate a significant (*p* < 0.001) correlation among the 121 pairs of reaction time and *d*´ ranks. Better reaction time ranks correlated with better *d*´ ranks on a participant-by-participant bases. At the group level, percussionists (Fig. 9, red dots, red long-dashed trendlines) tended to cluster toward the lower left corner of each plot, the location of best performance. By contrast, color guard (Fig. 9, blue dots, blue short-dashed trendlines) tended to cluster toward less favorable ranks in the top right corner of each plot. Brass players (Fig. 9, gold dots, medium-dashed trendlines) generally clustered between those two extremes, spanning the full rank range on reaction time and *d*´ alike. Overall, although each group generated at least a modestly positive trendline in each condition, the most positive slopes tended to occur within the color guard.

### Correlations between motion stimulus conditions

Figure 9 reveals considerable performance variation within each group. To determine the extent to which this within-group variation occurred nonrandomly, we constructed correlation matrices separately for each group. The first of these correlation matrices appears in Table 6, where cells reflect *d*´ rank correlations between pairs of motion stimulus conditions. A similar correlation matrix appears in Table 7, where the rank correlations pertain instead to reaction times. Visual inspection reveals positive and often strong rank correlations across groups and motion stimulus conditions, for *d*´ (Table 6) and reaction time (Table 7) alike. Moreover, the positive rank correlations reached statistical significance for all groups and conditions except one (color guard; radial-opposite-*d*´ by rotational-opposite-*d*´; *r*_(27)_ = 0.321, *p* = 0.09). Together, Tables 6 and 7 reveal significant consistent individual differences within groups, separate from the systematic between-group variation reported earlier in this section.

**Table 6.** Within-group rank correlations in *d*´

	Radial-opposite	Rotational-same	Rotational-opposite
Radial-same			
Percussion	0.785	0.758	0.696
Brass	0.755	0.880	0.875
Color guard	0.656	0.873	0.787
Radial-opposite			
Percussion		0.559	0.409
Brass		0.747	0.688
Color guard		0.502	0.321
Rotational-same			
Percussion			0.746
Brass			0.897
Color guard			0.851

For each group, cells indicate positive *d*´ rank correlations between pairs of motion–stimulus conditions. With the exception of the one condition (color guard; radial-opposite *d*´ by rotational-opposite *d*´ values; *r*_(27)_ = 0.321, *p* = 0.09) all correlations reached statistical significance (*p* < 0.05). The correlations demonstrate consistent individual differences in *d*´ ranks within each group.

**Table 7.** Within-group rank correlations in RT

	Radial-opposite	Rotational-same	Rotational-opposite
Radial-same			
Percussion	0.895	0.878	0.879
Brass	0.942	0.959	0.941
Color guard	0.717	0.810	0.779
Radial-opposite			
Percussion		0.879	0.887
Brass		0.933	0.898
Color guard		0.721	0.690
Rotational-same			
Percussion			0.917
Brass			0.969
Color guard			0.809

For each group, cells indicate positive RT rank correlations between pairs of motion–stimulus conditions. All correlations reached statistical significance (*p* < 0.05). The correlations demonstrate consistent individual differences in RT ranks within each group.

### Interaction between motion type and initial directions

**Figure 10.**
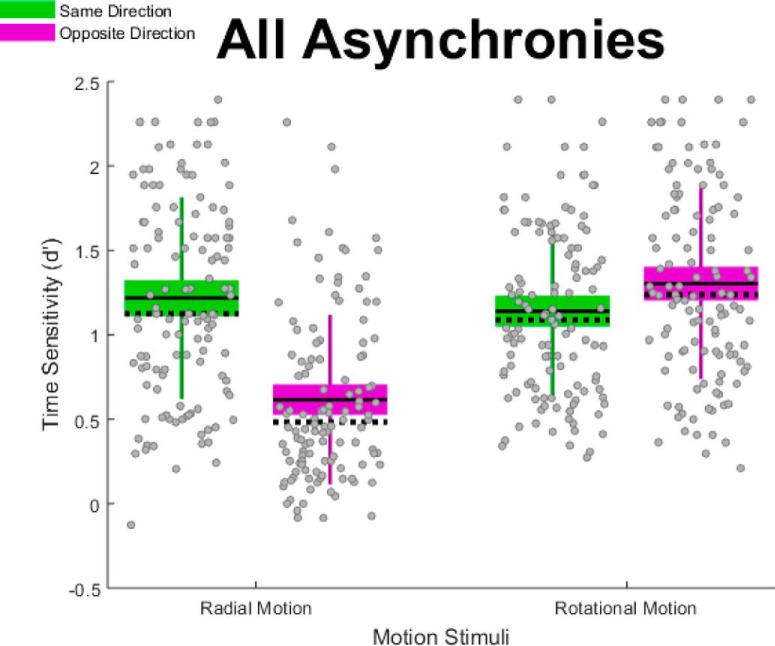
Motion type × initial directions interaction effect; descriptive statistics. Changing the initial directions from same (green boxes) to opposite (purple boxes) impaired TOJ precision (*d*´) in the radial motion condition. This pattern reversed when the motion type changed to rotational motion. Unlike standard box plots, each box extends upward and downward by 1 SD from the mean—the solid horizontal line centered within each box. The dotted horizontal line indicates the median, and the error bars indicate the 95% confidence interval. Gray dots correspond to individual data points.

In addition to analyzing the above-described group differences, we also analyzed the effect of our motion stimulus manipulations: motion type (radial, rotational) and initial directions (same, opposite). Figure 10 shows our primary motion stimulus finding—a significant two-way interaction between motion type and initial directions. Specifically, changing from same (Fig. 10, green boxes) to opposite (Fig. 10, purple boxes) initial directions impaired TOJs on radially defined asynchronies but improved TOJs on rotationally defined asynchronies. Although Figure 10 reflects data aggregated across all asynchronies, Movie 10 shows a similar crossover interaction at each temporal asynchrony.

Movie 10. Motion type by initial directions interaction effect; descriptive statistics. Changing the initial directions from same (green boxes) to opposite (purple boxes) impaired TOJ precision (*d*´) in the radial motion condition. This pattern reversed when the motion type changed to rotational motion. Unlike standard box plots, each box extends upward and downward by 1 SD from the mean—the solid horizontal line centered within each box. The dotted horizontal line indicates the median, and the error bars indicate the 95% confidence interval. Gray dots correspond to individual data points that collectively satisfied the normalcy assumption (Lilliefors test). Gray crosses correspond to individual data points that collectively failed to satisfy the normalcy assumption (Lilliefors test). 10.1523/ENEURO.0241-18.2018.video.10

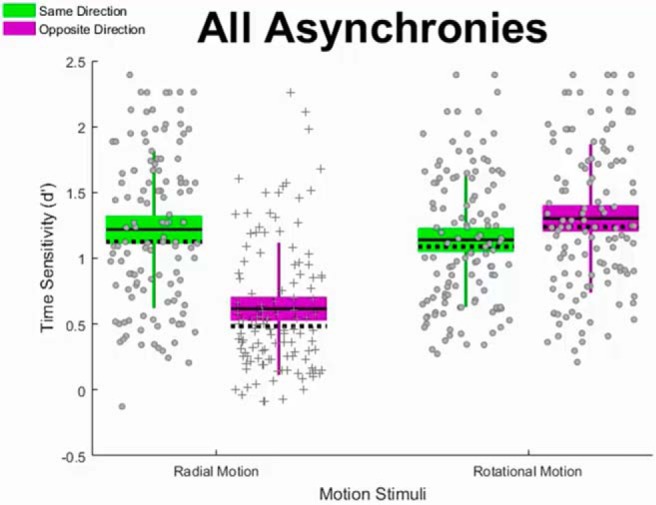

The data that generated the descriptive statistics in Movie 10 also generated the statistical analyses summarized in Table 8. For each combination of group and asynchrony, we computed the magnitude of the 2 × 2 motion type by initial directions interaction, expressed in *d*´ units. As described in the Materials and Methods, we evaluated this interaction effect using Monte Carlo simulations on medians rather than means because the data in some conditions failed to satisfy the normalcy assumption, per Lilliefors test. Briefly, the 2 × 2 interaction magnitude in each row of Table 5 reflects the following differencing computation among median *d*´ values. (Radial Same *d*´ − Radial Opposite *d*´) − (Rotational Same *d*´ − Rotational Opposite *d*´).

**Table 8:** **. Motion type by initial directions 2** × **2 Interaction effect; inferential statistics**

Group	Asynchrony	Interaction magnitude (*d*´)	Monte Carlo *p* value
All groups combined (*N* = 121)	All asynchronies combined	0.8019	<0.0001
	67 ms	0.8416	<0.0001
	133 ms	0.8788	<0.0001
	200 ms	1.214	<0.0001
Percussion (*N* = 25)	All asynchronies combined	0.8676	0.0005
	67 ms	1.078	<0.0001
	133 ms	0.825	0.0001
	200 ms	0.4851	0.0019
Brass (*N* = 67)	All asynchronies combined	0.7821	<0.0001
	67 ms	0.6469	<0.0001
	133 ms	0.8937	<0.0001
	200 ms	1.1193	<0.0001
Color guard (*N* = 29)	All asynchronies combined	0.7999	<0.0001
	67 ms	0.4176	0.0245
	133 ms	0.7701	0.0006
	200 ms	0.8579	0.0008

Each combination of group and asynchrony generated a significant motion type (radial, rotational) by initial directions (same, opposite) 2 × 2 interaction, according to nonparametric Monte Carlo simulations. Interaction magnitudes reflect *d*´ units. The significant 2 × 2 interactions across conditions argue against accounts in which shared neural events set the limit on radially and rotationally defined TOJs.

Significant 2 × 2 interactions in Table 8 disconfirm what one would predict if shared neural events set the limit on the precision of radially and rotationally defined TOJs.

## Discussion

The present study examined naturally occurring variability in a world class drum corps to provide new information about the temporal sensitivity of the visual system. Specifically, we investigated whether precise visual TOJs more strongly align with the color guard’s visual training or the musicians’ auditory training. The stimuli mimicked the radial and rotational asynchronies that typify the color guard’s visual performances. Despite this mimicry, the color guard exhibited significantly less precise visual TOJs than did brass players, who exhibited significantly less precise visual TOJs than did percussionists. Median visual TOJ thresholds spanned 10-fold, ranging between 29 ms (rotational opposite motion, percussion) and 290 ms (radial opposite motion, color guard). For context, the percussionists’ finest median TOJ threshold (29 ms; 34.48 Hz) reflects a temporal interval briefer than that between 32nd notes played at 180 beats/min (41.67 ms; 24 Hz). At the other extreme, the color guard’s largest median TOJ threshold (290 ms; 3.44 Hz) reflects a temporal interval longer than that between 8th notes played at 120 beats/min (250 ms; 4 Hz). This comparison highlights the visual time sensitivity differences between percussionists and the color guard in musical contexts (tempos) that typify Drum Corps International performances.

The reaction time data revealed that finer visual TOJ precision correlated significantly with faster, not slower, response speed. This disconfirmation of trade-offs between response precision and response speed occurred at the participant level and the group level. Across 121 participants, regression analyses within each motion stimulus condition indicated that better *d*´ ranks correlated significantly with better RT ranks (Fig. 9). At the group level, on median, the color guard responded 66 ms slower and less precisely than did brass players. On median, the brass players responded 74 ms slower and less precisely than did percussionists. Notably, in each of (7 asynchronies × 4 motion combinations) 28 experimental conditions, percussionists exhibited the briefest median RTs, followed by brass, then the color guard.

The musicians’ reaction time advantage over the color guard parallels previously reported reaction time advantages for musicians across diverse tasks. These tasks include spatial localization (Brochard et al., 2004), saccadic eye movements (Gruhn et al., 2006), and visual attention (Rodrigues et al., 2013). Moreover, among musicians the reaction time advantage, and the temporal smoothness of playing piano scales (Vaquero et al., 2016), correlate with musical training at an early age (Rodrigues et al., 2013). We did not collect information about the age at which our participants began musical training. However, our percussion and brass groups each reported median musical training durations of 8 years. Similarly, the color guard group reported a median of 7 years of spinning training. Given these comparable training durations across groups, it seems unlikely that training duration can explain our large groups differences in reaction time. Alternatively, the musicians’ reaction time advantage over the color guard might reflect greater oculomotor efficiency among musicians, perhaps arising from years of music reading practice (Gruhn et al., 2006). However, group differences in music reading practice would not explain percussionists’ reaction time advantage over brass players.

The percussionists’ reaction time advantage might reflect their unique responsibility to frequently divide musical tempos into very brief time units, such as 32nd notes. This possibility receives support from the SMS literature. Specifically, Hove et al. (2013) found greater SMS precision among musicians than among visual (video game and ball-sport) experts. Hove et al. (2013) explained the musicians’ superior synchronization performance by classifying musicians not merely as auditory experts, but rather as “auditory sensorimotor synchronization experts” (p. 396). By extension, one might classify DCI percussionists as 32nd note auditory SMS experts, and DCI brass players as 16th note auditory SMS experts. The difference between 32nd note and 16th note SMS expertise aligns with our percussionists’ performance advantage over our brass players. This framework could also explain our brass players’ performance advantage over our color guard participants. The color guard’s sensory motor synchronization rarely requires dividing musical tempos into units briefer than 8th notes. More importantly, unlike the auditory SMS expertise required of DCI percussionists and brass players, the DCI color guard require visual SMS expertise. The present work therefore connects with prior studies comparing timing sensitivity in the auditory versus visual modalities.

Numerous prior studies reveal better temporal performance for auditory stimuli than for visual stimuli. Examples include musical beat perception (Grahn, 2012), duration perception (Ortega et al., 2014), temporal interval production (Cicchini et al., 2012; Aagten-Murphy et al., 2014), temporal bisection thresholds (Cicchini et al., 2012), and synchronized finger tapping (Repp and Penel, 2002; Repp, 2005; Hove et al., 2013). However, recent reports (Iversen et al., 2015; Huang et al., 2017) have demonstrated comparably precise visual and auditory finger-tapping synchronization by replacing discretely flashed visual stimuli with continuous so-called “bouncing ball” stimuli. Presumably, the discrete visual flashes contain less tempo-related information than does the continuous vertical motion of a bouncing ball (Iversen et al., 2015; Huang et al., 2017).

The present study expanded on this continuous visual motion principle in two ways. First, we replaced the previously used continuous one-dimensional vertical motion (Iversen et al., 2015; Huang et al., 2017) with the continuous two-dimensional radial and rotational motion that color guard experts generate. Second, we tasked participants with TOJs that characterize the color guard’s expertise in generating precisely timed visual radial and rotational motion asynchronies. Despite the color guard’s expertise with asynchronies defined by continuous radial and rotational motion, the color guard exhibited significantly less precise TOJ thresholds than did musicians. The musician’s superior temporal precision echoes several prior studies (Cicchini et al., 2012; Rammsayer et al., 2012; Aagten-Murphy et al., 2014; Cameron and Grahn, 2014; Manning and Schutz, 2016; Slater and Kraus, 2016). Moreover, our finding of finer TOJ thresholds among percussionists than brass players parallels earlier work showing finer visual temporal bisection thresholds for percussionists than for string musicians (Cicchini et al., 2012).

The present findings also extend earlier perceptual learning reports that suggest auditory training enhances visual skills. For example, one study showed that unimodal visual motion detection improved significantly more from multimodal audiovisual training than from unimodal visual training (Seitz et al., 2006). A related experiment revealed that audio training and audiovisual training each significantly improved visual rhythm discrimination, but visual training did not (Barakat et al., 2015). This counterintuitive result aligns with a previously reported visual–auditory asymmetry in which auditory temporal discrimination training transferred to visual stimuli, but not vice versa (Bratzke et al., 2012).

Psychophysical experiments by Repp and Penel (2002) similarly have demonstrated three intriguing audiovisual asymmetries in SMS. Notably, all three of these SMS audiovisual asymmetries revealed auditory dominance even after participants received instructions to attend to visual rather than to auditory stimuli. First, participants finger-tapped more synchronously (less variably) to unimodal auditory stimuli than to unimodal visual stimuli. When researchers presented auditory and visual stimuli simultaneously, the precision of finger tapping matched that of the unimodal auditory condition and exceeded that of the unimodal visual condition. Second, auditory dominance emerged after researchers introduced a type of tempo-related “distractor” called an event onset shift (EOS). The EOS occurred when researchers experimentally hastened or slowed one auditory or visual pulse in an otherwise isochronous sequence that guided the participants’ finger tapping. The EOS generated corresponding hastening and slowing in finger tapping, a so-called phase correction response. However, the phase correction responses exhibited significantly tighter sensorimotor coupling to auditory than to visual EOSs. This finding foreshadowed subsequent experiments indicating that auditory distractors disrupt the discrimination of visually defined rhythms more than do visual distractors (Guttman et al., 2005). Third, after attempting to tap along with isochronous stimuli, participants also reported whether each stimulus sequence contained no EOS or an early or late EOS. Results showed significantly greater detection sensitivity for auditory than for visual EOSs. Stated differently, auditory EOSs remained subliminal (subthreshold) over a narrower temporal range than did visual EOSs. These three auditory-dominance findings (Repp and Penel, 2002) converge with our finding that auditory SMS experts (musicians) exhibited more precise visual TOJs than did visual SMS experts (the color guard).

Together, the psychophysically established audiovisual asymmetries summarized here merge well with methodologically diverse physiologic findings. For example, fMRI experiments have identified that the dorsal auditory pathway mediates rhythms trained in either the auditory modality or the visual modality (Karabanov et al., 2009). Consistent with this, transcranial magnetic stimulation (TMS) to the auditory cortex significantly disrupts auditory and visual time estimation, whereas TMS to the visual cortex disrupts visual time estimation only (Kanai et al., 2011). More recently, transcranial direct current stimulation experiments have shown that the posterior parietal cortex (PPC) mediates visual duration discrimination (Oyama, et al., 2017) and auditory–motor synchronization in professional drummers but not in nonmusicians (Pollok et al., 2017). This raises the possibility that the PPC facilitated our percussionists’ superior TOJs. Indeed, TOJs represent a type of temporal phase discrimination, and temporal phase discrimination declines after stimulating the PPC with continuous theta-burst TMS (Ross et al., 2018). Although the present psychophysical experiments do not permit precise physiologic conclusions, our findings point more strongly to the PPC than to middle temporal (MT) or medial superior temporal plus (MST^+^) areas. The evidence against a strong role for MT/MST^+^ comes from our radial and rotational motion dissociation, to which we now turn.

### Radial and rotational motion dissociation

For each group and each temporal asynchrony, we found a statistically significant and large interaction between initial directions and motion type. Specifically, changing from same to opposite initial directions impaired TOJs on radially defined asynchronies without impairing TOJs on rotationally defined asynchronies. The difference suggests a dissociation between the neural events that mediate radial versus rotational motion, a finding that is consistent with those of other recent experiments (Matthews et al., 2017). A dissociation seems surprising because monkey (Tanaka and Saito, 1989; Duffy and Wurtz, 1991a,b) and human (Smith et al., 2006; Gilmore et al., 2007; Wall et al., 2008; Strong et al., 2017) physiologic data indicate that radial and rotational motion register in a shared brain region: area MST+.

**Figure 11.**
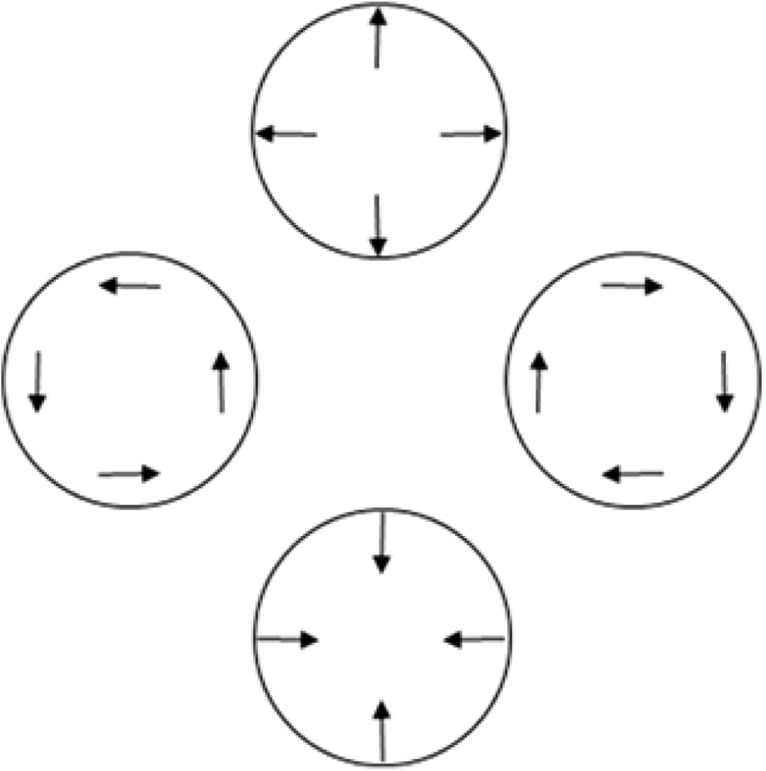
Relationships among radial, rotational, and linear motion. Rotating the local linear motion signals (arrows) 90º changes global radial motion to global rotational motion, and vice versa. Cortical neurons in the MST^+^ complex respond to each of these motion types.

The dissociation also disconfirms the parsimonious possibility that the present TOJs reflect how precisely participants registered local linear components in our radial and rotational stimuli (Koenderink and van Doorn, 1976; Freeman and Harris, 1992; Clifford et al., 1999; Barraza and Grzywacz, 2002, 2005; Wurfel et al., 2005; Yu et al., 2010). Figure 11 schematizes this computational principle. Figure 11 shows the local linear components (Fig. 11, arrows) for radial expansion (Fig. 11, top), clockwise rotation (Fig. 11, right), radial contraction (Fig. 11, bottom), and anticlockwise rotation (Fig. 11, left). Note that simply rotating the local linear motion components through 90° changes the global motion from rotational to radial, and vice versa. Local linear components of radial and rotational motion register in the MT brain region (Fig. 2, red region; Albright, 1984). MT innervates the neighboring radially and rotationally sensitive MST^+^ complex (Fig. 2, blue region; Albright, 1984). Therefore, one could expect radial and rotational motion sensitivity to reflect how precisely these shared local linear signals register in MT or combine in MST^+^. Indeed, the precision with which MT and MST^+^ register or combine local linear motion cues could have set the limit on TOJ precision in the present study. This possibility predicts comparable TOJ precision across our initial directions and motion-type conditions over which local linear motion signals remained constant. Contrary to this prediction, TOJ precision depended significantly on the initial directions by motion-type interaction. This interaction disconfirms the possibility that the MT/MST^+^ brain regions critically limited the precision on our radial and rotational motion TOJs.

What else might have generated the observed dissociation between radial and rotational motion? One possible explanation originates from an earlier finding that looming (radially expanding) stimuli capture attention but receding (radially contracting) stimuli do not (Franconeri and Simons, 2003). To the extent that looming stimuli captured attention more than receding stimuli, one would expect worse TOJ performance for opposite than for same radial directions. This difference would arise because correct TOJ responses in our study required precisely registering both the left and right direction changes. Attending to one side while ignoring the other would impair performance on this inherently relational (TOJ) task. Alternatively, or in addition, heightened attention to looming targets could have hastened the neural relay to the cortex, creating response biases toward the looming stimulus. Attentionally driven response biases would cause suboptimal TOJ performance. Critically, attentional capture by looming stimuli would generate errors in the opposite-direction radial condition but not in the same-direction radial condition. Moreover, attentional capture by looming stimuli would not pertain to any of our rotational motion conditions. An impairment specific to the opposite-direction radial condition matches our data. In any case, future psychophysical experiments could pinpoint the source of this large, reliable, and unpredicted dissociation between radial and rotational motion.

### Within-group consistent individual differences

Future psychophysical experiments also could pinpoint the source of the large, reliable, and unpredicted within-group consistent individual differences observed here. Specifically, within each group, participants who responded more precisely and faster tended to do so significantly across stimulus conditions (Tables 6, 7). These consistent individual differences could reflect a combination of factors. Possibilities include differences in training histories, expertise level, and lapse rates (e.g., motor errors or errors from inattention).

Our significant correlations across stimulus conditions additionally suggest future research on an issue raised in the visually bouncing-ball experiment in the study by Iversen et al. (2015). Iversen et al. (2015) reported that, although participants exhibited comparable finger-tapping precision to auditory stimuli and visually bouncing balls, only the auditory stimuli generated “metrical perception.” Metrical perception refers to how participants subjectively organize (“meter”) the temporal structure of periodic stimuli. As an example, so-called subjective accenting occurs when we perceptually group a sequence of physically identical sounds (“tick-tick-tick-tick”) into pairs (“TICK-tock-TICK-tock”). Perceptual pairs, or other perceptual groupings, would presumably generate subtle but systematic finger-tapping irregularities that emerge at higher-order (greater than one) lags in autocorrelation analyses. Using autocorrelations as a window into perceptual organization, Iversen et al. (2015) found distinct autocorrelation patterns when participants tapped to auditory versus visually bouncing stimuli. Specifically, for participants with typical hearing abilities, auditory stimuli generated higher-order (lag 3) autocorrelations but visually bouncing balls did not. Remarkably, in contrast to typical hearing participants, deaf participants exhibited significant higher-order (lag 3) autocorrelations to visual stimuli, suggesting beat perception. Deaf participants also exhibited significantly greater SMS to visual stimuli than hearing participants. This raises the possibility that the ability of deaf participants to extract a beat from visual stimuli generated their SMS advantage over the hearing participants. Stated more broadly, experts organize relevant information more effectively than do novices; metrical perception could serve as an information-organizing mechanism that confers performance advantages. Accordingly, future autocorrelation analyses on DCI musicians and color guard might determine whether metrical perception explains the group differences and consistent individual differences observed here.

### Conclusion

World class drum corps performances require cooperation among color guard members, brass players, and percussionists to produce highly precise SMS. Despite this shared demand, we observed statistically significant and large median group differences in visual timing sensitivity within a world class drum corps. The quasi-experimental nature of our study (nonrandom assignment to groups) suggests at least two possible nonexclusive causes for the observed group differences: self-selection and plasticity (Chopin et al., 2017). In principle, subtle pre-existing differences in visual timing sensitivity may have predisposed drum corps members to self-select their roles in color guard, brass, or percussion. Alternatively, or in addition, visual and/or auditory plasticity may have permitted years of group-specific training demands to generate large group differences in visual timing sensitivity. In any case, seizing on years of naturally occurring training histories affords a unique window into sensory systems and skills underlying world class performance art.

## Synthesis

Reviewing Editor: Satu Palva, University of Helsinki Decisions are customarily a result of the Reviewing Editor and the peer reviewers coming together and discussing their recommendations until a consensus is reached. When revisions are invited, a fact-based synthesis statement explaining their decision and outlining what is needed to prepare a revision will be listed below. The following reviewer(s) agreed to reveal their identity: Aleksi Sihvonen, John Iversen.

Synthesis:

Both reviewers found that your study was interesting and well designed in investigating using a bilateral visual radiating and rotating plaid pattern stimuli mimicking the color guard's visual displays, whether the precise visual temporal order judgments (TOJs) were more improved by color guard's visual training or musicians' auditory training. The focus on color guard and drum corps members was found to be valuable natural experimental population. Participants were 25 percussionists, 67 brass players and 29 color guards from a world class drum corps. The results show that the percussionists exhibited significantly lower TOJ thresholds than the brass players, and that the brass players exhibited significantly lower TOJ thresholds than the color guards. The results suggest that extensive auditory timing training has a stronger effect of visual timing than does visual training

Both reviewers also found several suggestions on how to improve your manuscript. The essential changes required by the reviewers are described below. Additionally, presenting multiple conditions as movies maybe disadvantage relative to traditional multi-panel plots as many readers are using PDFs where the movies are not accessible. Hence, the movie should be complemented by a a classical figure that is accessible to all readers.

As one of the major issues, it was found that the conceptual stage for this work should be set more broadly, drawing e.g. from relevant literature in the timing and synchronization fields, which has a long history of studying the relationship and limits of timing abilities in different modalities. More specifically, the findings should be conceptually bound to work by Bruno Repp (see Repp BH, Su Y-H (2013)). Also some discussion of how and if the current temporal order judgment relates to other timing abilities, such as synchronization, which is central to the drum corps performance needs would be needed to set the stage in the introduction (See Psychonomic Bul Rev 20:403-53.). Why it is important to test both radial and rotational order judgments, and the conceptual significance of same vs. opposite tasks. Do you expect them all to behave similarly, or are they probing important mechanistic differences?.

For those not familiar with drum corps, a detailed descriptive paragraph about a performance and the timing demands of each group would be very useful.

What are the alternative explanations that can explain the results for the observed group differences. In musicians, the neuroplastic changes in the brain are associated with the age of musical training onset (Vaguero et al. 2016 Neuroimage), and musicians who began their musical practice earlier present better reaction times in visual attention tests (Rodrigues et al. 2013 Brain Cogn). Similarly, adult musicians have been shown to have enhanced visual-spatial abilities in previously published articles (e.g. Brochard et al. 2004 Brain Cogn), and the effect seems to be most consistent in visual test reaction times, possibly due to music reading practice and more efficient oculomotor strategies in musicians (e.g. Gruhn et al. 2006 Music Sci). In this light, the training history and the age of onset of musical training should be controlled. This information is not available and could easily be included in a background variable table. If this information is available for the authors, did the musicians have more training years than the color guards? Is it possible that color guards had not trained as long as the percussionists and brass players? If the information on age of onset of musical training and color guard training is available, it would be interesting to see if the age of onset of training is associated with even lower TOJ thresholds (within group).

Moreover, is it possible that their performance can be more tolerant of timing deviations than e.g. drumming and thus does not require timing accuracy as strongly?

An additional important question is are the color guard members as a group are differently talented than drummers and therefore some information about this selection history would be relevant (for example if color guard members are drawn from those who fail auditions for other parts, or not). Did color guard members come from a musical background. There are many opportunities for early musical training, but fewer for early color guard training, so is it possible that the drummers are simply more experienced?

Please briefly address the extent to which the test stimuli really simulate the timing demands of color guard members.

Here are additional references it seems worthwhile to consider in the discussion

Other references related to auditory/visual timing:

Grahn JA (2012) See what I hear? Beat perception in auditory and visual rhythms. Exp Brain Res 220:51-61.

Hove MJ, Iversen JR, (null), Repp BH (2013) Synchronization with competing visual and auditory rhythms: bouncing ball meets metronome. Psych Res 77:388-98.

The work of Yi-Huang Su, in general. e.g. Su Y-H (2016) Visual tuning and metrical perception of realistic point-light dance movements. Sci Rep 6:22774.

Other studies of superior timing in percussionists

Slater J, Kraus N, Woodruff Carr K, Tierney AT, Azem A, Ashley R (2018) Speech-in-noise perception is linked to rhythm production skills in adult percussionists and non-musicians. Language, Cognition and Neuroscience 33:710-7.

Manning FC, Schutz M (2015) Trained to keep a beat: movement-related enhancements to timing perception in percussionists and non-percussionists. Psych Res 1-11.

Cameron DJ, Grahn JA (2014) Enhanced timing abilities in percussionists generalize to rhythms without a musical beat. Front Hum Neurosci

Other studies of TMS/PPC

Ross JM, Iversen JR, Balasubramaniam R (2018) The Role of Posterior Parietal Cortex in Beat-based Timing Perception: A Continuous Theta Burst Stimulation Study. J Cogn Neurosci 30:634-43.

Also several methodological issues were raised which are detailed below:

More detailed information about the groups and participants are needed including distribution of the degree of experience in each group (years of practice, any other measures of the quality of their skill--such as their ranking within the group), and demonstration to what extent other factors (gender, age, other musical experience, video gaming) are balanced across groups or not, and why they are relevant/irrelevant.

Why the choice to test after an intense rehearsal? Could that have (differentially) impacted results? Is color guard more physically demanding?

Pease explain the reason for the assumptions on line 189 & 191. Is this standard practice? Does the exact value impact the results?

Was RT and d' correlated on a subject-by-subject basis?

There is considerable variation in the results within groups. Is there any way to understand this? Is it correlated across conditions within individuals? Does it relate to any measures of experience or proficiency at their musical craft? At least comment on it is needed.

The transparency of indicating lillefore's test within the plots is visually distracting. It would be preferable if all individual points were drawn the same, and the legend indicate which conditions passed/failed. It is important, but of secondary importance, and thus not perhaps a good use of the reader's limited attention.

line 442 expanding--&gt;expanded

Minor comments:

1) Abstract, line 14: “29 color guard”, change to color guards. Similarly, line 110. Insert empty space to line 27: “... -superior temporal...”.

2) It seems that the font size changes in various places, please correct (e.g. lines 80-81 and 302).

3) Line 164: The experiment was carried out after all participants had completed a 13-hour drum corps rehearsal. Did all the participants have the same amount of practice or did some group (e.g. color guards) practice more hours than percussionists?

4) While acknowledging the authors' statement on lines 480-481: “However, we refrain from drawing precise physiological conclusions from our psychophysical data”, I would have liked to see some brief neuroanatomical explanation in the Discussion why the MST and MT areas can be developed by musical training.
